# Calcium silicate induces mitophagy-mediated metabolic shifts toward oxidative phosphorylation in BMSCs to facilitate osteogenesis and bone regeneration

**DOI:** 10.1093/rb/rbaf101

**Published:** 2025-10-01

**Authors:** Yu Qiu, Jun Tian, Yaxin Lou, Xiaoqian Yang, He Liu, Chunfeng Pang, Yuhua Xiong, Mengjie Li, Weiyang Chen, Qian Tao, Ya Shen, Xi Wei

**Affiliations:** Hospital of Stomatology, Guanghua School of Stomatology, Sun Yat-Sen University, Guangzhou, Guangdong 510055, P.R. China; Guangdong Provincial Key Laboratory of Stomatology, Guangzhou, Guangdong 510055, P.R. China; Hospital of Stomatology, Guanghua School of Stomatology, Sun Yat-Sen University, Guangzhou, Guangdong 510055, P.R. China; Guangdong Provincial Key Laboratory of Stomatology, Guangzhou, Guangdong 510055, P.R. China; Hospital of Stomatology, Guanghua School of Stomatology, Sun Yat-Sen University, Guangzhou, Guangdong 510055, P.R. China; Guangdong Provincial Key Laboratory of Stomatology, Guangzhou, Guangdong 510055, P.R. China; Hospital of Stomatology, Guanghua School of Stomatology, Sun Yat-Sen University, Guangzhou, Guangdong 510055, P.R. China; Guangdong Provincial Key Laboratory of Stomatology, Guangzhou, Guangdong 510055, P.R. China; Division of Endodontics, Department of Oral Biological and Medical Sciences, The University of British Columbia, Vancouver, British Columbia V6T 1Z3, Canada; Hospital of Stomatology, Guanghua School of Stomatology, Sun Yat-Sen University, Guangzhou, Guangdong 510055, P.R. China; Guangdong Provincial Key Laboratory of Stomatology, Guangzhou, Guangdong 510055, P.R. China; Hospital of Stomatology, Guanghua School of Stomatology, Sun Yat-Sen University, Guangzhou, Guangdong 510055, P.R. China; Guangdong Provincial Key Laboratory of Stomatology, Guangzhou, Guangdong 510055, P.R. China; Hospital of Stomatology, Guanghua School of Stomatology, Sun Yat-Sen University, Guangzhou, Guangdong 510055, P.R. China; Guangdong Provincial Key Laboratory of Stomatology, Guangzhou, Guangdong 510055, P.R. China; Hospital of Stomatology, Guanghua School of Stomatology, Sun Yat-Sen University, Guangzhou, Guangdong 510055, P.R. China; Guangdong Provincial Key Laboratory of Stomatology, Guangzhou, Guangdong 510055, P.R. China; Hospital of Stomatology, Guanghua School of Stomatology, Sun Yat-Sen University, Guangzhou, Guangdong 510055, P.R. China; Guangdong Provincial Key Laboratory of Stomatology, Guangzhou, Guangdong 510055, P.R. China; Division of Endodontics, Department of Oral Biological and Medical Sciences, The University of British Columbia, Vancouver, British Columbia V6T 1Z3, Canada; Hospital of Stomatology, Guanghua School of Stomatology, Sun Yat-Sen University, Guangzhou, Guangdong 510055, P.R. China; Guangdong Provincial Key Laboratory of Stomatology, Guangzhou, Guangdong 510055, P.R. China

**Keywords:** calcium silicate, bone marrow mesenchymal stem cells, bone regeneration, mitochondria, oxidative phosphorylation

## Abstract

Calcium silicate (CS)-based bioactive materials were widely utilized to promote the therapeutic potential of bone marrow mesenchymal stem cells (BMSCs) in bone tissue engineering. The activation of numerous classic bone formation modulators, including the BMP, Wnt, and MAPK/ERK signaling pathways, contributes to the CS-induced osteogenesis of BMSCs. Mitochondrial metabolic patterns have emerged as key contributors to the osteogenic differentiation of mesenchymal stem cells. However, whether CS affects the mitochondrial metabolic profiles of BMSCs is mostly unclear. Herein, we showed that CS induced the osteogenic differentiation of human BMSCs (hBMSCs) mainly via silicon (Si) ion release. Moreover, CS-stimulated hBMSCs underwent metabolic reprogramming accompanied by increased mitochondrial oxidative phosphorylation (OXPHOS) activity. The inhibition of OXPHOS hindered the CS-induced osteogenic differentiation of hBMSCs and bone regeneration, indicating that CS-induced OXPHOS mediated the observed increase in osteogenesis. Mechanistically, CS induced mitophagy and autophagic flux by increasing the formation of autolysosomes and lysosomal degradation to eliminate dysfunctional mitochondria and mitochondrial reactive oxygen species production, leading to enhanced OXPHOS and osteogenesis in hBMSCs. Furthermore, CS promoted mitochondrial fusion in hBMSCs, which may contribute to OXPHOS activation. Our investigation reveals a previously unclear function of CS in regulating the osteogenesis of BMSCs by inducing mitophagy-mediated metabolic shifts toward OXPHOS.

## Graphical abstract

**Figure:**
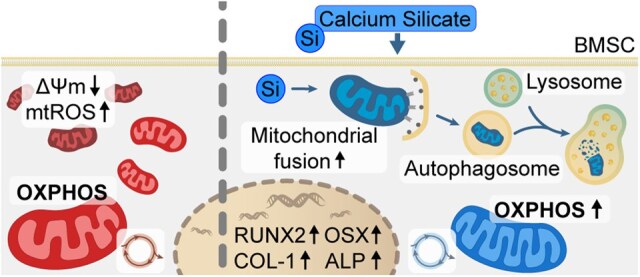

## Introduction

Bone tissue engineering is considered an excellent, feasible solution for treating large bone defects exceeding the critical size. Although autografts are defined as the gold standard for bone repair, donor site injury, the risk of disease transmission, and the inflammatory response limit their application [1]. The emergence of cell-friendly bioactive materials has contributed greatly to advancing cell-based bone tissue engineering strategies, and these materials have illustrated satisfactory outcomes both *in vitro* and *in vivo* [2, 3]. However, to date, most studies have been confined to assessing the bone regeneration capacity of the materials and their shortcomings. Prior to transitioning from laboratory studies to clinical development, exploring the interactions between biomaterials and mesenchymal stem cells (MSCs) is crucial for elucidating the biological behavior of materials for more rational and appropriate applications.

Recently, methods for regulating MSC metabolic profiles have been combined with the use of biomaterial scaffolds to provide a promising cell-based strategy to accelerate bone regeneration via precise regulation of energy metabolism [4, 5]. The specific metabolic profiles of MSCs differ under certain microenvironments and play vital roles in determining the fate of MSCs during differentiation. Numerous types of undifferentiated stem cells, including MSCs, depend on glycolysis for energy demand. By reducing oxygen tension, glycolysis can help preserve the stemness of proliferating stem cells [6, 7]. Throughout the osteogenic differentiation of MSCs, the oxygen consumption rate (OCR) and intracellular adenosine triphosphate (ATP) level significantly elevate with the activation of mitochondrial oxidative phosphorylation (OXPHOS). The metabolic change from glycolysis to OXPHOS maximizes energy generation to meet high ATP demands and biosynthetic demands during osteogenesis [8, 9]. In contrast, impaired mitochondrial OXPHOS induced by respiratory chain complex inhibitors (e.g. antimycin A (AA)), uncouplers (e.g. FCCP), or ATP synthase inhibitors (e.g. oligomycin (Oligo)) in MSCs causes compromised osteogenic differentiation [10–12]. Therefore, unveiling the inﬂuence of biomaterials on the mitochondrial metabolic profile of MSCs might facilitate the design and synthesis of innovative biomaterials for bone tissue engineering [4, 13].

Calcium silicate (CS)-based bioactive glasses and ceramics were extensively investigated for bone regeneration. Their key advantage lies in the release of calcium (Ca) and especially silicon (Si) ions, which promotes MSC proliferation and osteogenic differentiation [14–16]. Earlier investigations illustrated that Si ions can promote osteogenesis via several classic bone formation-modulating pathways, including the BMP, Wnt, Shh, and MAPK/ERK signaling pathways [17–19]. However, whether CS or Si ions affect the metabolic profile of MSCs to determine their osteogenic differentiation is largely unknown. Herein, we concentrated on the detailed function of CS in mitochondrial metabolism in MSCs and showed that Si ions released from CS can promote mitophagy, through which dysfunctional mitochondria are eliminated to induce OXPHOS, thus leading to enhanced osteogenesis of human bone marrow stem cells (hBMSCs). Our study highlights the role of mitochondrial metabolism in defining the MSCs’ fate via the utilization of bioactive materials and leads to the development of better strategies for cell engineering using these materials and MSCs.

## Materials and methods

### Cell culture

The ScienCell Company (San Diego, USA) was the source of primary hBMSCs. The cells were cultivated and expanded in alpha minimum essential medium (α-MEM; Invitrogen, USA) enriched with 15% fetal bovine serum (FBS; Gibco, USA), 100 U/ml penicillin and 100 μg/ml streptomycin (Invitrogen, USA) at 37°C in a humidified 5% CO_2_ incubator. hBMSCs at passages 3-8 were utilized for subsequent analyses.

### Characterization of hBMSCs

The mesenchymal phenotype of the hBMSCs was confirmed as per the criteria recommended by the International Society of Cellular Therapy (ISCT). Suspended hBMSCs were collected and stained with antibodies specific for CD73/90/105/34/45 (PE-conjugated antihuman antibodies; eBioscience, CA, USA) for 30 min in darkness at 4°C. Expression profiles were assessed using a ﬂow cytometer system (BD Bioscience, San Jose, CA, USA).

### Preparation of CS extracts

For the preparation of CS extracts, CS powder (1 g) (Kunshan, China) was eluted in 50 ml of α-MEM at 37°C for 24 h. The extracts were subsequently collected and filtered via a sterile 0.22-μm microfiltration membrane. Various dilutions (final dilutions: 50%, 25%, 12.5% and 6.25%) of the medium extracts were then prepared in α-MEM.

### Ion release analysis

Si, Ca, and phosphorus (P) ion levels in the CS extracts were assessed via inductively coupled plasma optical emission spectroscopy (ICP-OES) (iCAP 6500 Duo; Thermo Fisher Scientific, Florence, KY, USA).

### Cell growth assay

For cell growth assessment, hBMSCs were cultivated in 96-well plates (3 × 10^3^ cells/well, *n* = 5) in the presence or absence of various CS extract dilutions. After 1, 2, and 3 days in culture, Cell Counting Kit (CCK)-8 reagent was applied to a 96-well plate (10 μl/well) (Dojindo Molecular Technology; Japan), which was subsequently incubated at 37°C for 2 h. The optical density (OD) at 450 nm was subsequently assessed via an enzyme-linked immunosorbent assay (ELISA) plate reader.

### Osteogenic differentiation assay

hBMSCs were cultured in a 6-well plate with or without 25% CS extract for 24 h. To induce osteogenic differentiation, the medium was substituted with osteogenic medium (OM) containing 100 µM L-ascorbic acid 2-phosphate, 2 mM β-glycerophosphate, and 10 nM dexamethasone (all from Sigma-Aldrich). After 7 days, hBMSCs were rinsed with PBST (PBS +0.05% Tween-20) and fixed with 4% paraformaldehyde (PFA) for 2 min at room temperature (RT). Then, cell incubation was conducted with BCIP/NBT staining solution (Stemgent, USA) for 15–30 min at RT. Purple staining indicated alkaline phosphatase (ALP)-positive cells. After 14 days, matrix mineralization was evaluated via 1% alizarin red staining (ARS; Sigma-Aldrich) for 30 min at RT, and mineralized nodules were imaged.

### Real-time polymerase chain reaction

Total RNA isolation from cultivated hBMSCs was conducted via the RNA-Quick Purification Kit (Yishan, China) per the manufacturer’s protocol. cDNA synthesis was conducted via the PrimeScript™ RT Reagent Kit (TaKaRa, Japan). Real-time polymerase chain reaction was conducted with Fast SYBR Green Master Mix (Thermo Fisher, USA) and gene-specific primers (Supplementary Table S1). The normalization of mRNA levels to β-actin was conducted via the 2^–ΔΔCT^ method.

### Mitochondrial profile analysis

hBMSCs were stimulated with 25% CS extract for 24 h. MitoTracker Green, MitoTracker Red CMXRos (to assess the mitochondrial membrane potential), and MitoSOX (to detect mtROS) staining were subsequently conducted as per the manufacturer’s guidelines (Invitrogen, USA). The mitochondrial profile of hBMSCs was estimated via a ﬂow cytometer system (BD Bioscience, CA, USA).

### Western blotting

Total protein isolation from hBMSCs was conducted via RIPA lysis buffer with protease and phosphatase inhibitors, and protein levels were assessed via a BCA assay kit (all from Thermo Fisher Scientific, USA). For WB, separation of 20 μg of protein was conducted by SDS-PAGE (Invitrogen, USA) and transferred to 0.2 μm PVDF membranes (Millipore, USA). 5% bovine serum albumin (BSA) and 0.1% Tween-20 (Sigma, USA) were utilized to block the membranes for 1 h at RT, then an overnight incubation was conducted at 4°C with primary antibodies against human targets: LC3I/II (Cell Signaling Technology, Beverly, USA), p62 (Santa Cruz Biotechnology, Dallas, USA), and β-actin (Sigma-Aldrich, USA), and the latter was used as a loading control. A 1-h incubation of the membranes was conducted at RT with species-specific horseradish peroxidase (HRP)-conjugated secondary antibodies (1:10 000; Cell Signaling Technology). Immunoreactive bands were visualized via enhanced chemiluminescence (ECL) reagents (Millipore, USA).

### Lentiviral transfection

Transfection of hBMSCs was conducted with a lentiviral plasmid encoding mCherry-GFP-LC3B (pLenti-CMV-mCherry-GFP-LC3B-IRES-Puro, OBiO Technology) to evaluate autophagic flux. Autolysosomes (mCherry-positive, red) and autophagosomes (mCherry/GFP double-positive, yellow) were detected via a laser-scanning confocal microscope (Zeiss LSM 980, Germany).

### Immunofluorescence staining

For immunostaining of translocase of the outer membrane 20 (Tom20) and/or lysosomal-associated membrane protein 1 (LAMP1), hBMSCs (2 × 10^3^ cells/well) were plated on chamber slides (Nunc, NY, USA). Subsequently, 4% PFA was utilized to fix the cells for 15 min, 0.1% Triton X-100 to permeabilize them, and 5% BSA to block them. The incubation of slides was conducted with primary antibodies overnight at 4°C and followed by incubation with Alexa Fluor 488- or 568-conjugated secondary antibodies (1:200, Invitrogen) for 1 h at RT. Lastly, the mounting of slides was conducted via Vectashield mounting medium with 4',6-diamidino-2-phenylindole (DAPI; Abcam, USA).

For immunostaining of osteopontin (OPN) and ALP, hBMSCs (1.5 × 10^3^ cells/well) were seeded on chamber slides and then induced to undergo osteogenic differentiation for 4 days. After fixation, permeabilization, and blocking of nonspecific sites, the incubation of cells was conducted with anti-OPN/ALP primary antibodies (1:300; Affinity, Jiangsu, China) and then with Alexa Fluor 649-conjugated secondary antibodies for 1 h at RT. The cytoskeleton was stained with ActinGreen 488 ReadyProbes (Thermo Fisher Scientific, USA). The slides were mounted using Vectashield mounting medium with DAPI, and observed under a confocal microscope (Zeiss LSM 980, Zeiss, Germany). The mean fluorescence intensity (MFI) was quantified using Image J software.

### Extracellular flux analysis

hBMSCs were treated with or without 25% CS extract for 24 h and seeded into Seahorse XF96 microplates at 8 × 10^4^ cells/well. Before the assay, cells were rinsed and kept at 37°C in a non-CO_2_ incubator with XF base medium (Seahorse; 102353; Agilent) enriched with 2 mM L-glutamine and 2 mM sodium pyruvate (Gibco) for 1 h. Real-time OCR measurements were performed using an XF-96 Analyzer (Seahorse Bioscience) following sequential injection of Oligo (1 mM), FCCP (0.5 mM), and rotenone (Rot; 100 nM) plus AA (1 mM) (all from Sigma–Aldrich). Basal respiration equaled the OCR recorded prior to the first injection minus the lowest OCR observed after the final injection (nonmitochondrial oxygen consumption). ATP production was derived from basal OCR minus post- Oligo OCR. Maximal respiratory capacity (MRC) was calculated as peak OCR following FCCP administration minus nonmitochondrial OCR, while spare respiratory capacity was calculated as maximal respiration minus basal respiration.

### Metabolomics analysis

After 24 h of stimulation with 25% CS extract, hBMSCs were collected by scraping and centrifugation. Cell pellets (1 × 10^7^ hBMSCs) were sent to the Beijing Genomics Institute (BGI) for metabolite profiling via LC–MS/MS in positive and negative ion modes. Data from both modes were merged, and duplicate metabolites were eliminated. The resulting dataset was analyzed using MetaboAnalyst 5.0 (McGill University, Canada). Principal component analysis (PCA) and heatmaps were generated using the “Statistical Analysis” tool, and metabolite set enrichment analysis (MSEA) was conducted via the “Enrichment Analysis” tool.

### Transmission electron microscopy

Transmission electron microscopy (TEM) was performed to assess mitochondrial morphology and the formation of autophagosomes and autolysosomes. Briefly, hBMSCs were treated with or without 25% CS extract for 24 h in complete αMEM. The collected cells were fixed with 2.5% glutaraldehyde and sliced into ultrathin sections. A Tecnai G2 spirit Twin transmission electron microscope (Czech Republic) was utilized to assess the sections at 100 kV.

### Animals

Male BALB/c nude mice (18–21 g, 6–8 weeks old) were acquired from the Laboratory Animal Center of Sun Yat-Sen University (Guangzhou, China), with each animal serving as a single experimental unit. Mice were housed in specific pathogen-free circumstances at 23°C under a 12-h light/dark cycle with unrestricted access to food and water. The Institutional Animal Care and Use Committee of Sun Yat-Sen University gave its approval for each procedure (No. SYSU-IACUC-2023-000185).

### Subcutaneous transplantation

Porous HA/β-TCP scaffolds (HA: TCP = 6:4, 4 mm diameter and 2 mm depth; National Engineering Research Center for Biomaterials, Chengdu, China) with pore sizes of 100–500 μm and 75% porosity were used as cell carriers. Control hBMSCs (5 × 10^5^) or hBMSCs subjected to different treatments (5 × 10^5^) were dropped onto each β-TCP scaffold under sterile conditions and cultured *in vitro* for 24 h. After using 1% sodium pentobarbital (50 mg/kg) to anesthetize the nude mice, 20 mice were allocated to the following 4 groups (*n* = 5) in a random manner using a random number generator to ensure unbiased group assignment: (1) the control group, in which scaffolds seeded with untreated hBMSCs were applied; (2) the CS group, in which scaffolds seeded with CS-treated hBMSCs were applied; (3) the CS+Oligo group, in which scaffolds seeded with hBMSCs treated with the OXPHOS inhibitor Oligo in the presence of CS were applied; and (4) the CS+BafA1 group, in which scaffolds seeded with hBMSCs treated with the lysosomal inhibitor bafilomycin A1 (BafA1) in the presence of CS were applied. A 5–10 mm longitudinal incision was created in the skin using sterile surgical scissors. Then, the subcutaneous tissue was bluntly dissected with forceps to form a small pocket. The cell-loaded scaffolds were subsequently transplanted subcutaneously into the BALB/c nude mice. After eight weeks, the mice were euthanized, and the scaffolds were collected and fixed in 4% PFA. No animals were excluded from this experiment.

### Calvarial bone defect surgery

Control hBMSCs or hBMSCs subjected to different treatments (CS-treated hBMSCs, CS+Oligo-treated hBMSCs, or CS+BafA1-treated hBMSCs) were seeded onto PLGA scaffolds (Shandong Academy of Pharmaceutical Sciences, Shandong, China) for 24 h *in vitro*. 30 mice were allocated in a random manner to the following 5 groups (*n* = 6) using a random count generator to ensure unbiased group assignment: (i) the negative control (NC) group, in which scaffolds without cells were applied; (ii) the control group, in which scaffolds seeded with untreated hBMSCs were applied; (iii) the CS group, in which scaffolds seeded with CS-treated hBMSCs were applied; (iv) the CS+Oligo group, in which scaffolds seeded with hBMSCs treated with the OXPHOS inhibitor Oligo in the presence of CS were applied; and (v) the CS+BafA1 group, in which scaffolds seeded with hBMSCs treated with the lysosomal inhibitor BafA1 in the presence of CS were applied. After anesthetization with 1% sodium pentobarbital (50 mg/kg), an incision was made in the midline of the mouse cranium. Full-thickness defects (a 4-mm diameter) were formed using a dental drill on the right side of the mouse skull via saline irrigation, and cell-loaded scaffolds or scaffolds without cells (for the NC group) were implanted into the defects of BALB/c nude mice. The mice were sacrificed 8 weeks post-surgery, and their craniums were collected and fixed in 4% PFA. Subsequently, if a nude mouse died, if the incision or implanted scaffold caused skin ulceration and erosion, or if the incision failed to heal, the sample from that particular nude mouse was eliminated from the study. Herein, one nude mouse in the CS+BafA1 group exhibited an unhealed incision, and the scaffold was ejected; this mouse was excluded from further study and was humanely euthanized with an overdose of sodium pentobarbital.

### Micro-computed tomography analysis

Fixed specimens were subjected to Micro-computed tomography (micro-CT; μCT50, Scanco Medical, Switzerland) scanning and evaluation. All the samples were scanned at 70 kV and 114 mA, with a spatial resolution of 10 μm. Thereafter, volumetric three-dimensional (3D) reconstructions and histomorphometric analysis were conducted with built-in software. The bone volume fraction (BV/TV) was calculated within the regions of interest. The analysis was conducted by an investigator blinded to the group allocation.

### Histological analysis and immunofluorescence staining of subcutaneous implants and calvarial bone

After decalcification in 10% EDTA for 2 weeks at RT, samples were paraffin-embedded and sectioned at 5 μm. Hematoxylin and eosin (HE) staining was conducted to assess bone structure, and Masson’s trichrome staining evaluated collagen maturity. Sections were scanned using a slice scanning microscope (Aperio AT2, Leica, Germany). Immunofluorescence staining employed goat anti-rabbit ALP (1:150, Affinity, Jiangsu, China) and anti-rabbit OPN (1:150, Affinity) antibodies. Nuclei were counterstained with DAPI, and images were observed via confocal microscopy (Zeiss LSM 980, Germany). Analyses were conducted by an investigator blinded to group distribution.

### Statistical analysis

Statistical analyses were conducted via GraphPad Prism 9 (USA). Data are represented as mean ± standard deviation (SD). Parametric comparisons were performed using Student’s *t* test or one-way analysis of variance (ANOVA). When the overall ANOVA was significant, pairwise comparisons between groups were performed using Bonferroni *post hoc* tests. The Kruskal–Wallis rank sum test was applied for data with unequal variations. *P *< 0.05 was deemed significant.

## Results

### Si ions release is indispensable for the capability of CS to induce the osteogenic differentiation of hBMSCs

**Figure 1.**
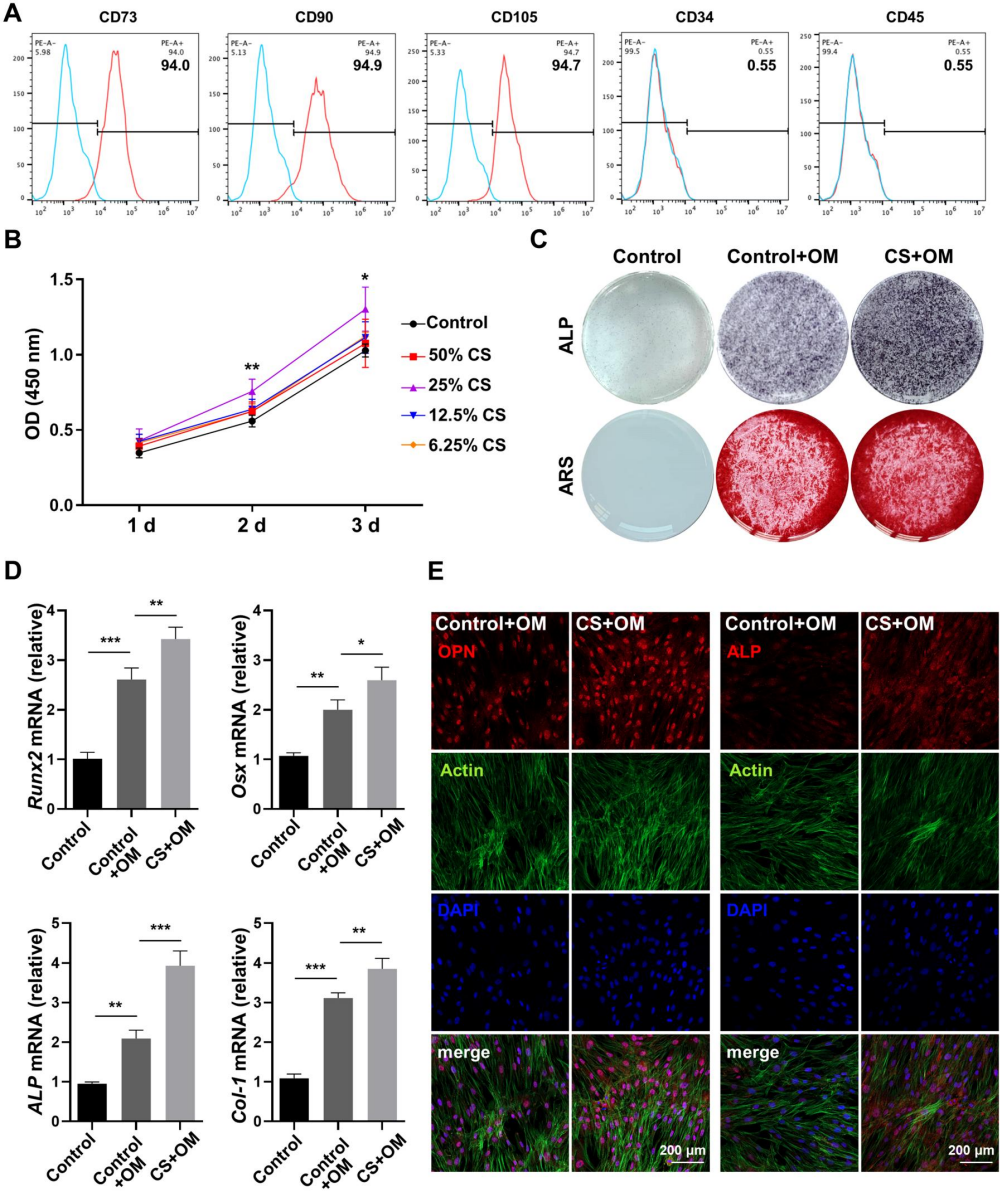
CS induces the osteogenic differentiation of hBMSCs. (**A**) The percentages of hBMSCs positive for CD73/90/105/34/45 were determined using flow cytometry. (**B**) The proliferation of hBMSCs exposed to various CS extract dilutions was evaluated via the CCK-8 (*n* = 5, *P* < ** 0.01, * 0.05, 25% CS vs. control). (**C**) ALP staining at 7 days post-osteogenic induction and ARS staining at 14 days post-osteogenic induction in hBMSCs treated with 25% CS extract. (**D**) The relative mRNA levels of *Runx2*, *Osx*, *ALP,* and *Col-1* in hBMSCs treated with 25% CS extract at 7 days post-osteogenic induction (*n* = 3). (**E**) Immunofluorescence staining of OPN and ALP in hBMSCs treated with 25% CS extract at 4 days after osteogenic induction. The error bars signify the mean ± SD. *P* < ***0.005; ** 0.01; * 0.05.

Flow cytometry demonstrated that up to 94% of the cells were positive for MSC surface markers, including CD73/90/105, but negative for the hematopoietic markers CD34/45, with 0.55% of the cells expressing these markers (Figure 1A). Thus, the cultured cells possessed a mesenchymal phenotype. To explore the impacts of CS extracts on the growth of hBMSCs, we cultivated cells in 50%, 25%, 12.5% or 6.25% CS extracts for 24, 48 or 72 h. The CCK-8 revealed no significant variation in cell growth after 24 h, although cell growth slightly increased after culture with various dilutions of CS extract. Interestingly, the groups treated with 25% CS extract exhibited greater proliferation than did the control and other groups after culture for 48 or 72 h (Figure 1B). Therefore, 25% was selected as the optimal level for the subsequent experiments. After 7 days of culture under osteogenic conditions, hBMSCs treated with 25% CS extract had significantly greater ALP activity, accompanied by upregulated mRNA expression of osteogenic markers, including *Runx2*, *Osx*, *ALP* and *Collagen 1* (*Col-1*), than did the control hBMSCs (Figure 1C and D). Furthermore, compared to the control+OM group, the CS+OM group presented a higher ARS intensity, indicating enhanced matrix mineralization post-osteogenic induction for 14 days (Figure 1C). Immunofluorescence staining further confirmed the elevated expression of OPN and ALP in CS+OM-treated cells (Figure 1E and Supplementary Figure S1A).

Moreover, CS can release large numbers of Ca and Si ions to trigger the osteogenic differentiation of BMSCs. Herein, the concentrations of Si (105.7 mg/L) and Ca (171.3 mg/L) ions were greater in the CS extract than in the α-MEM (less than 1 mg/L and 70.3 mg/L, respectively) (Table 1). However, the addition of CaCl_2_ (69.4 mg/L) to α-MEM, resulting in a Ca ion concentration identical to that in the 25% CS extract, did not significantly enhance the osteogenic differentiation of hBMSCs, as indicated by ALP and ARS staining (Supplementary Figure S1B). Moreover, the addition of CaCl_2_ upregulated only the *Col-1* expression without altering the levels of *Runx2*, *Osx* or *ALP* (Supplementary Figure S1C). These data indicate that, in our study, CS induced the osteogenic differentiation of hBMSCs, possibly mainly *via* Si ion release.

**Table 1.** Ion (Si, Ca, and P) concentrations (mg/L) of the CS extracts

	Si	Ca	P
α-MEM	0.63 ± 0.20	70.25 ± 1.56	33.96 ± 0.80
CS	105.70 ± 0.90**	171.30 ± 2.21**	4.09 ± 0.05**

** *P *< 0.01 compared to the data for the same ion in α-MEM.

### CS-treated hBMSCs commit to OXPHOS

**Figure 2.**
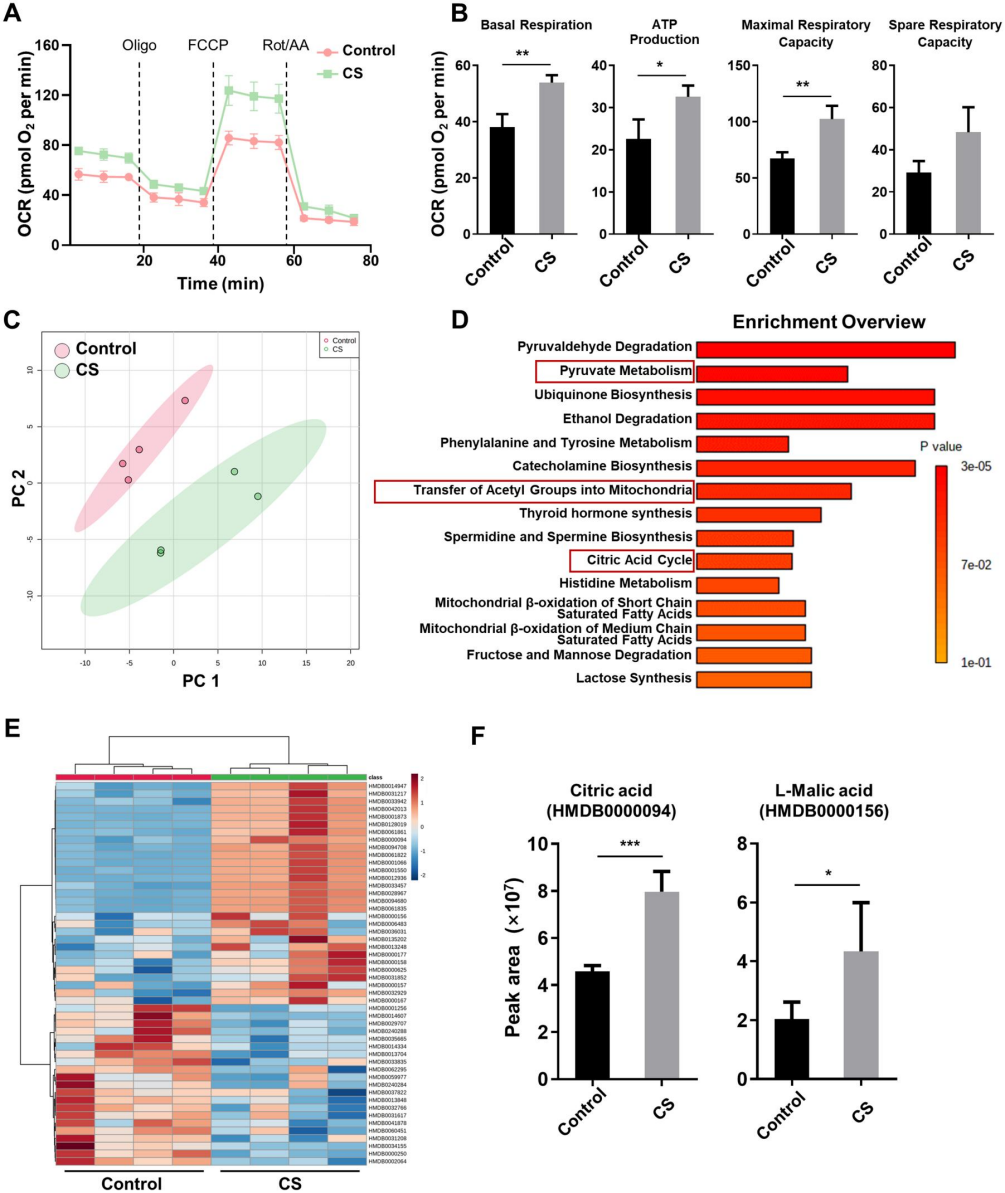
CS-treated hBMSCs exhibit enhanced OXPHOS activity and metabolic reprogramming. hBMSCs were cultured with or without CS extract for 24 h. (**A**) OCR was assessed at baseline and subsequent sequential injection of Oligo, FCCP, and rotenone plus antimycin A (Rot/AA) (*n* = 3). (**B**) Basal respiration, ATP generation, MRC, and spare respiratory capacity were quantified by measuring OCR (*n* = 3). (**C**) PCA of metabolomics data from control and CS-treated hBMSCs (*n* = 4). (**D**) MSEA of differentially abundant metabolites between groups was performed using MetaboAnalyst. (**E**) Heatmap showing the 50 most significantly affected metabolites in hBMSCs stimulated with CS extract. (**F**) Peak areas of citric acid cycle metabolites in the control and CS-treated hBMSCs according to mass spectrometry (*n* = 4). The error bars signify the mean ± SD. *P* < ***0.005; ** 0.01; * 0.05.

Mitochondrial OXPHOS is vital for the osteogenic differentiation of MSCs [20]. We thus explored the bioenergetic profiles of hBMSCs treated with or without CS extract by measuring the OCR. Our results revealed greater OXPHOS activity, such as basal respiration, ATP generation, and MRC, in the CS-treated hBMSCs than in the control hBMSCs. However, the CS-treated hBMSCs did not exhibit significantly greater spare respiration than the control hBMSCs did (Figure 2A and B). To assess global metabolic changes, a comprehensive LC–MS/MS-based metabolomic analysis was performed on CS-treated and control hBMSCs (the original data are included in the Supplementary Materials). We verified that the metabolic profile of the CS-treated hBMSCs, which presented increases in the concentrations of the citric acid cycle metabolites, citric acid and L-malic acid, was distinct from that of the control hBMSCs (Figure 2C, E and F). MSEA demonstrated the enrichment of metabolites related to the OXPHOS pathway, such as pyruvate metabolism, transfer of acetyl groups into mitochondria and the citric acid cycle, in the CS-exposed hBMSCs (Figure 2D). Consistent with OCR analysis, MSEA further suggested metabolic commitment to OXPHOS in the CS-stimulated hBMSCs.

To further confirm whether the OXPHOS induction by CS contributes to the promoted BMSC osteogenic differentiation, we used the OXPHOS inhibitor Oligo. Oligo caused a significant decrease in the OCR in the CS-stimulated hBMSCs (Figure 2A). Furthermore, the capability of CS to trigger the osteogenic differentiation of hBMSC decreased in the presence of Oligo, as indicated by decreased ALP and ARS staining intensities (Supplementary Figure S2A), as well as decreased expression of *Runx2*, *Osx*, *ALP* and *Col-1* (Supplementary Figure S2B). Similarly, compared to that in the CS group, the protein expression of OPN and ALP in the CS+Oligo group was downregulated, as shown by decreased immunofluorescence staining intensity (Supplementary Figure S2C and D). Collectively, these outcomes illustrate that CS promotes the osteogenesis of hBMSCs by inducing their metabolic preference for OXPHOS.

### CS promotes mitophagy in hBMSCs

**Figure 3.**
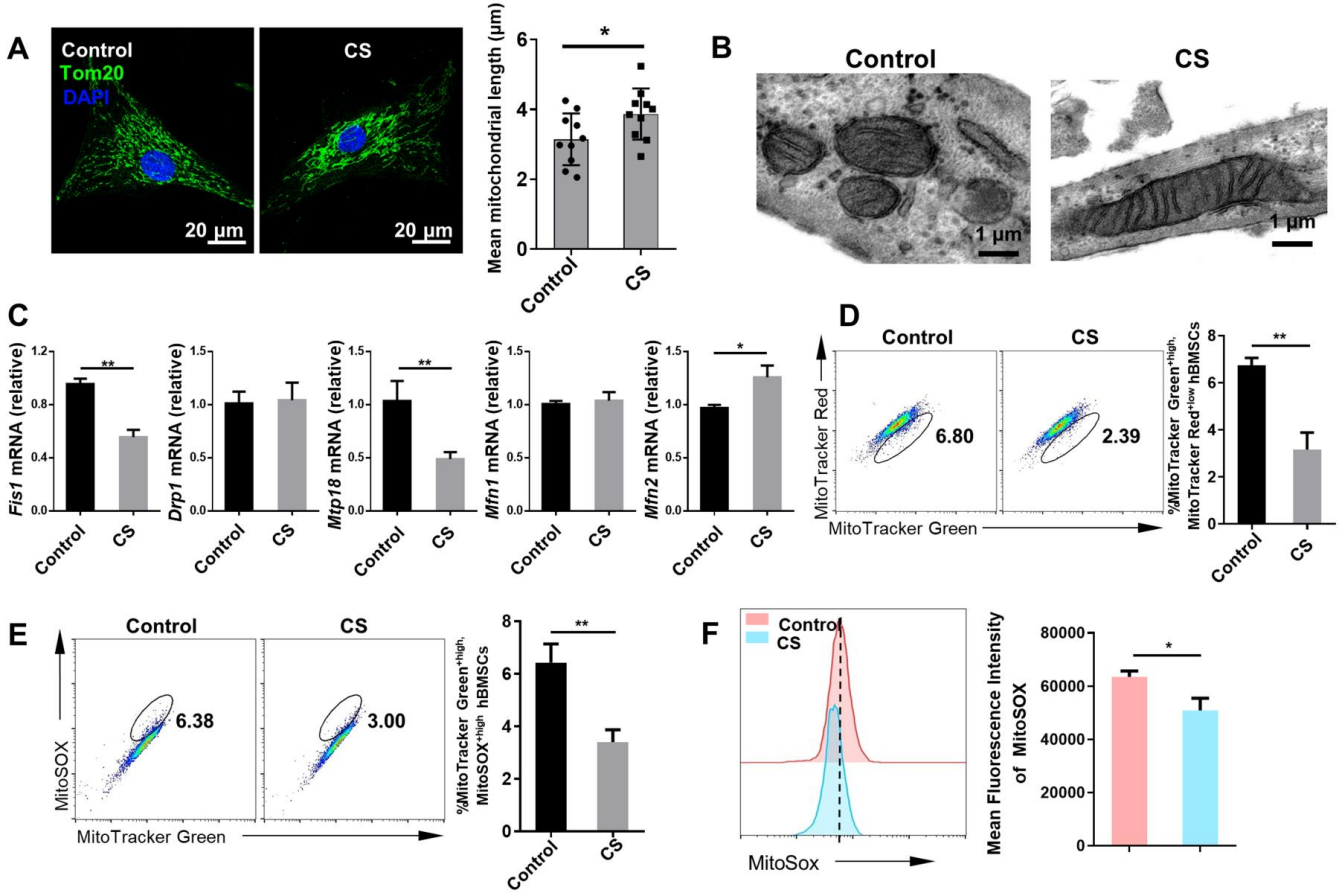
CS promotes mitochondrial fusion and eliminates dysfunctional mitochondria in hBMSCs. (**A**) The morphology of mitochondria labeled with an anti-Tom20 antibody (green) in the control and CS-treated hBMSCs was observed using laser-scanning confocal microscopy. The mean mitochondrial length was recorded (*n* = 10). (**B**) TEM image illustrating the morphology of mitochondria in control and CS-treated hBMSCs. (**C**) The relative mRNA levels of the mitochondrial fission/fusion-associated factors *Fis1*, *Drp1*, *Mtp18*, *Mfn1,* and *Mfn2* in the control and CS-treated hBMSCs (*n* = 3). (**D**) Control and CS-treated hBMSCs were labeled with the ΔΨm-independent probe MitoTracker Green and the ΔΨm-sensitive probe MitoTracker Red. The proportion of dysfunctional mitochondria was analyzed (MitoTracker Green^+high^, MitoTracker Red^+low^) (*n* = 3). (**E**) Labelling the control and CS-treated hBMSCs was conducted with MitoTracker Green and the mitochondria-specific ROS probe MitoSOX. The percentage of hBMSCs producing mtROS (MitoTracker Green^+high^, MitoSOX^+high^) was determined (*n* = 3). (**F**) MitoSOX fluorescence intensity was measured to quantify mtROS levels in hBMSCs (*n* = 3). The error bars signify the mean ± SD. *P* < ** 0.01; *0.05.

The structure of mitochondria is highly plastic and dynamic, and mitochondria undergo both fusion and fission procedures. Mitochondrial dynamics affect the efficiency of OXPHOS. Mitochondrial fragmentation can impair OXPHOS, whereas mitochondrial fusion enhances OXPHOS levels [21, 22]. Thus, we investigated whether the morphology of mitochondria in hBMSCs is modulated by CS. We observed that compared with control hBMSCs, the CS-treated hBMSCs had elongated mitochondria, as illustrated by immunofluorescence staining of Tom20 and TEM (Figure 3A and B). Moreover, after CS treatment, the mRNA levels of the mitochondrial fission-associated factors mitochondrial fission protein 1 (Fis1) and mitochondrial protein 18 (MTP18) were decreased, whereas those of the mitochondrial fusion-related factor mitofusin 2 (Mfn2) were increased in hBMSCs. Nevertheless, no significant alterations were shown in the levels of dynamin-related protein 1 (Drp1) or Mitofusin 1 (Mfn1) (Figure 3C). These results demonstrate that CS can induce mitochondrial fusion in hBMSCs.

Next, to further analyze the mitochondrial profile of hBMSCs, we used the mitochondrial membrane potential (ΔΨm)-independent probe MitoTracker Green in combination with the ΔΨm-sensitive probe MitoTracker Red [23]. According to flow cytometry, CS treatment reduced the percentage of dysfunctional mitochondria in hBMSCs (MitoTracker Green^+high^, MitoTracker Red^+low^) (Figure 3D). Loss of ΔΨm in polarized mitochondria causes a high mitochondrial reactive oxygen species (mtROS) generation [24, 25]. Using the mitochondria-specific ROS probe MitoSOX, we detected a lower proportion of mtROS-producing hBMSCs (MitoTracker Green^+high^, MitoSOX^+high^) in the CS group than in the control group (Figure 3E). Moreover, mtROS levels were lower in the CS-treated hBMSCs than in the control hBMSCs, as shown by flow cytometry (Figure 3F). We also found that the CS-treated hBMSCs had fewer dysfunctional mitochondria and decreased mtROS levels in the presence of OM (Supplementary Figure S3A and B). Our results indicate that CS can eliminate dysfunctional mitochondria and mtROS production in hBMSCs.

**Figure 4.**
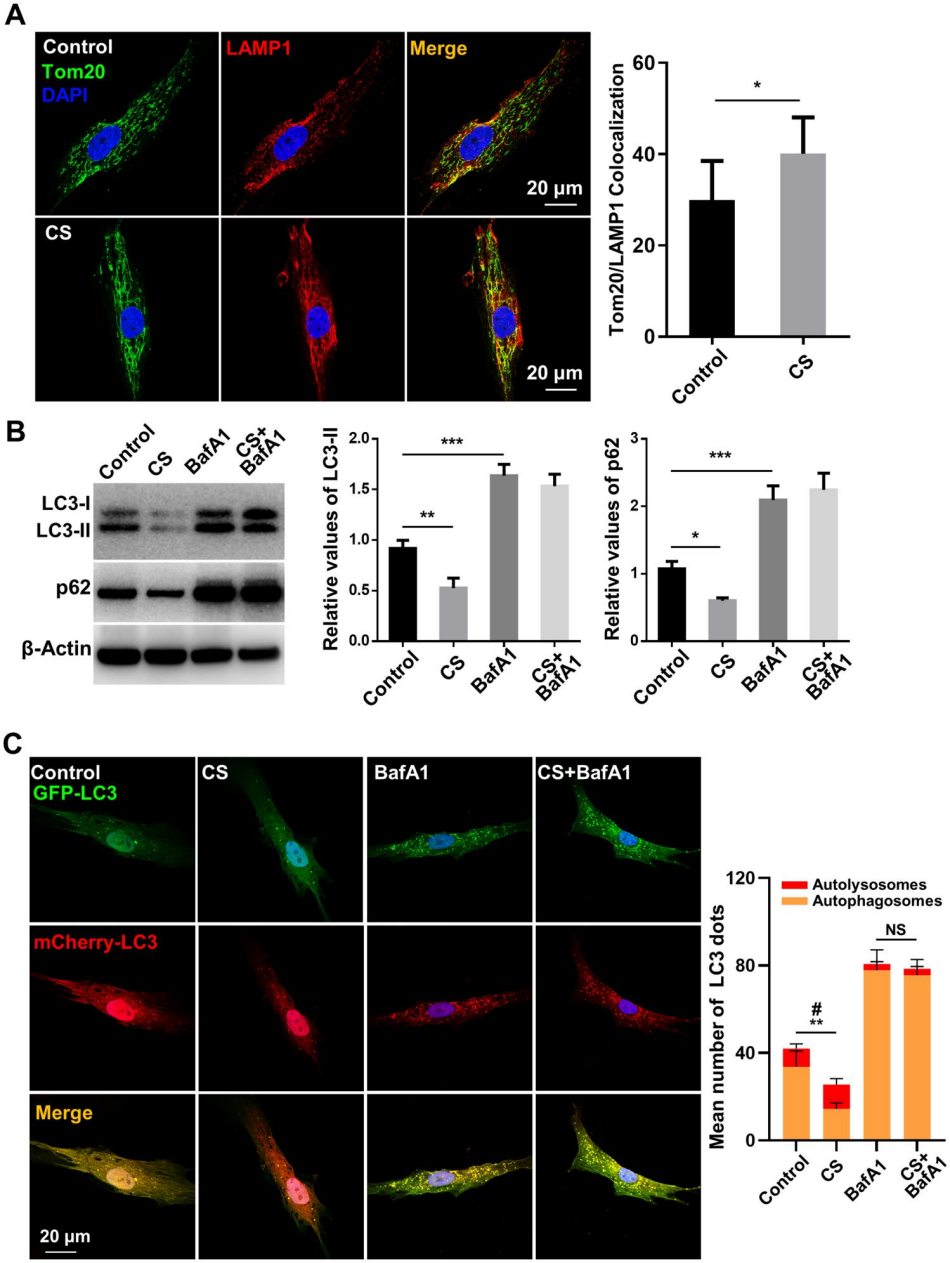
CS induces mitophagy and autophagic flux in hBMSCs. (**A**) Colocalization of the mitochondrial marker Tom20 and lysosomal marker LAMP1 was observed via confocal microscopy in control and CS-treated hBMSCs. (**B**) hBMSCs were treated with CS extract for 12 h, with or without BafA1 (100 nM; applied 2 h prior to harvest). LC3-I/II and p62 levels were assessed by WB and quantified relative to β-actin (*n* = 3). Uncropped blots are shown in Supplementary Figure S6. (**C**) hBMSCs transfected with mCherry-GFP-LC3B were analyzed for autophagic flux after 12 h of CS treatment. The right panel illustrates the LC3 puncta quantification (*n* = 10, **P < 0.01 for total puncta; ^#^*P* < 0.05 for red puncta). Data are reported as mean ± SD. *P* < *** 0.005; **0.01; * 0.05.

As a specialized type of autophagy, mitophagy is vital for mitochondrial quality control through the targeted removal of dysfunctional or excessive mitochondria [26]. To verify whether CS promotes mitophagy to eliminate dysfunctional mitochondria in hBMSCs, we stained the mitochondria and lysosomes of hBMSCs with Tom20 (green) and LAMP1 (red), respectively. Colocalization of mitochondria and lysosomes displayed a significant elevation in CS-treated hBMSCs compared to untreated hBMSCs, indicating enhanced mitophagy (Figure 4A). To assess whether CS induces autophagic flux (including autophagosome formation, fusion with lysosomes, and the eventual autophagic substances degradation), hBMSCs were exposed to the lysosomal inhibitor BafA1 in the presence of CS. BafA1 specifically inhibits vacuolar-type H^+^-ATPase (V-ATPase), a proton pump that maintains the acidic pH of intracellular organelles, including lysosomes, and the extracellular environment. Its ability to block lysosomal acidification and subsequent protein degradation makes it a widely used tool for evaluating autophagic flux [27, 28]. Intriguingly, WB analysis illustrated that CS treatment suppressed LC3-II and p62 expression (Figure 4B). LC3-II suppression may indicate either suppressed autophagy or increased lysosomal degradation. The blockage of autophagic flux with BafA1 resulted in similarly elevated LC3-II and p62 levels in both control and CS-treated hBMSCs (Figure 4B), suggesting that CS enhances lysosomal clearance rather than autophagosome formation. To further assess autophagic flux, hBMSCs were transduced with a lentiviral mCherry-GFP-LC3B construct. Compared to controls, CS-treated hBMSCs showed a higher ratio of red puncta (autolysosomes, as GFP is quenched in acidic lysosomes) and fewer total LC3 puncta, indicating increased autolysosome formation and elevated autophagic flux. In accordance with WB data, BafA1 inhibited GFP degradation in both control and CS groups, as evidenced by similar LC3 puncta counts (Figure 4C). We further observed the cell ultrastructure using TEM. Both autophagosomes and autolysosomes appeared in the cytoplasm of control hBMSCs, indicating the occurrence of early and late stages of mitophagy. In contrast, a markedly increased number of autolysosomes was observed in the CS-treated group, whereas few autophagosomes were present (Supplementary Figure S4). These data demonstrate that CS may promote the fusion of autophagosomes with lysosomes, eventually resulting in accelerated lysosomal degradation and elevated autophagic flux in hBMSCs.

**Figure 5.**
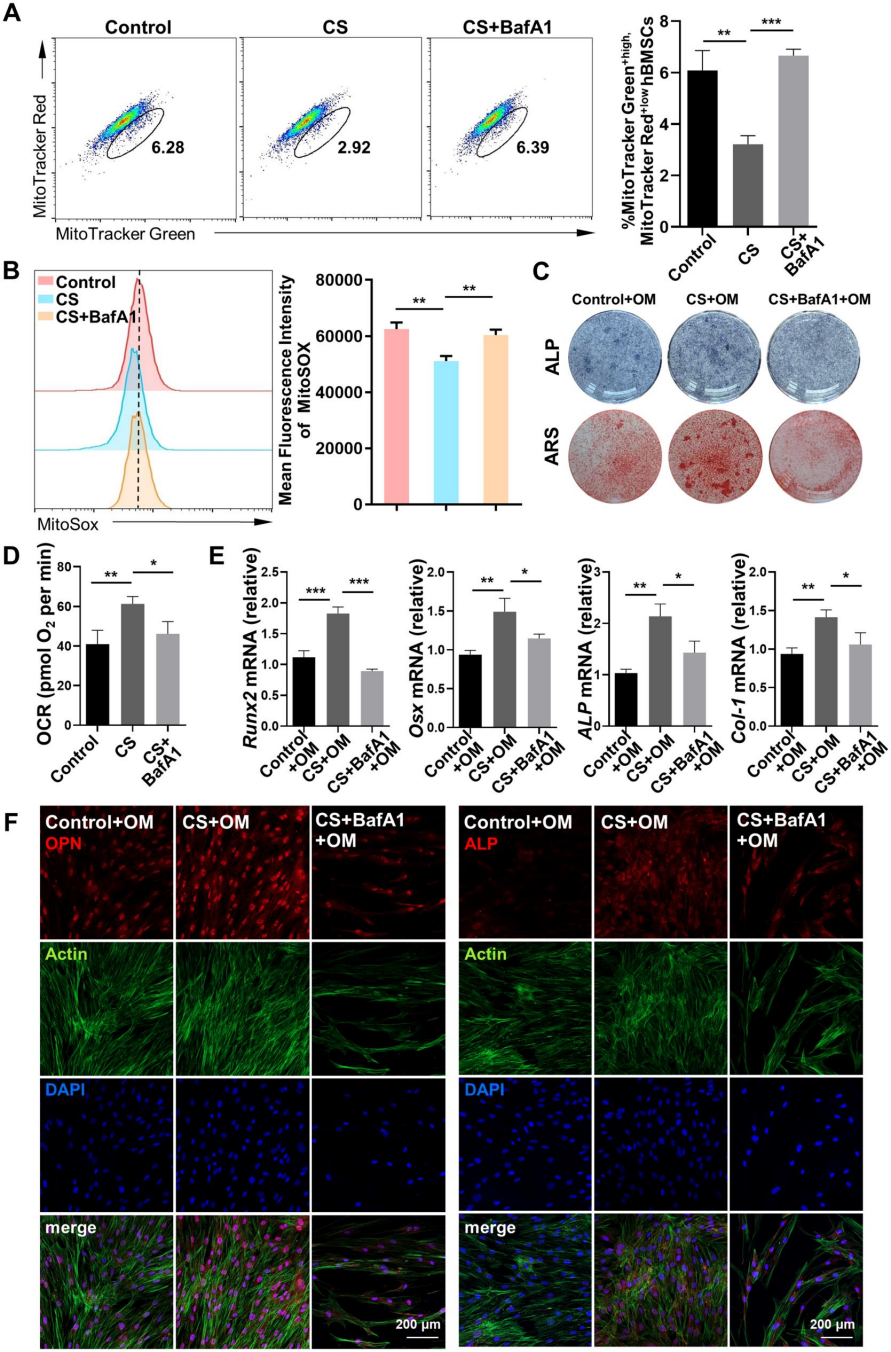
CS-induced mitophagy promotes OXPHOS activity. (**A**) hBMCSs stimulated with CS in the presence of BafA1 were labeled with the probes MitoTracker Green and MitoTracker Red. The percentage of dysfunctional mitochondria was analyzed (*n* = 3). (**B**) mtROS levels in hBMSCs stimulated with CS in the presence of BafA1 were assessed by measuring the MitoSOX fluorescence intensity (*n* = 3). (**C**) ALP staining at 7 days after osteogenic induction and ARS staining at 14 days post-induction in hBMSCs treated with CS and BafA1. (**D**) The basal OCR was assessed in hBMSCs exposed to CS with or without BafA1 (*n* = 3). (**E**) The relative mRNA levels of *Runx2*, *Osx*, *ALP*, and *Col-1* in hBMSCs exposed to CS or BafA1 at 7 days after osteogenic induction (*n* = 3). (**F**) Immunofluorescence staining of OPN and ALP in hBMSCs exposed to CS with or without BafA1 at 4 days subsequent to osteogenic induction. The error bars denote the mean ± SD. *P* < *** 0.005; ** 0.01; * 0.05.

Mitophagy can reprogram stem cell differentiation by modulating the metabolic profile [29]. Our above findings prompted us to further explore whether CS-induced mitophagy promotes OXPHOS to enhance the osteogenesis of hBMSCs. We found that repressing the formation of autolysosomes by BafA1 impaired CS-mediated mitophagy in hBMSCs, as indicated by increased dysfunctional mitochondria, with or without OM (Figure 5A and Supplementary Figure S3A). Furthermore, when hBMSCs were exposed to BafA1, CS treatment failed to decrease mtROS production (Figure 5B and Supplementary Figure S3B) or increase the OCR (Figure 5D). CS also showed a decreased ability to promote osteogenic differentiation in the presence of BafA1, as indicated by its impaired ability to increase the ALP and ARS staining intensity or the expression of *Runx2*, *Osx*, *ALP* and *Col-1* (Figure 5C and E). Moreover, the immunofluorescence staining intensities of OPN and ALP were decreased in the existence of BafA1 (Figure 5F and Supplementary Figure S3C). These findings indicate that CS induces the osteogenic differentiation of hBMSCs by promoting the mitophagy-mediated metabolic transition toward OXPHOS.

### CS-induced mitochondrial OXPHOS enhances the osteogenic capacity of hBMSCs *in vivo*

**Figure 6.**
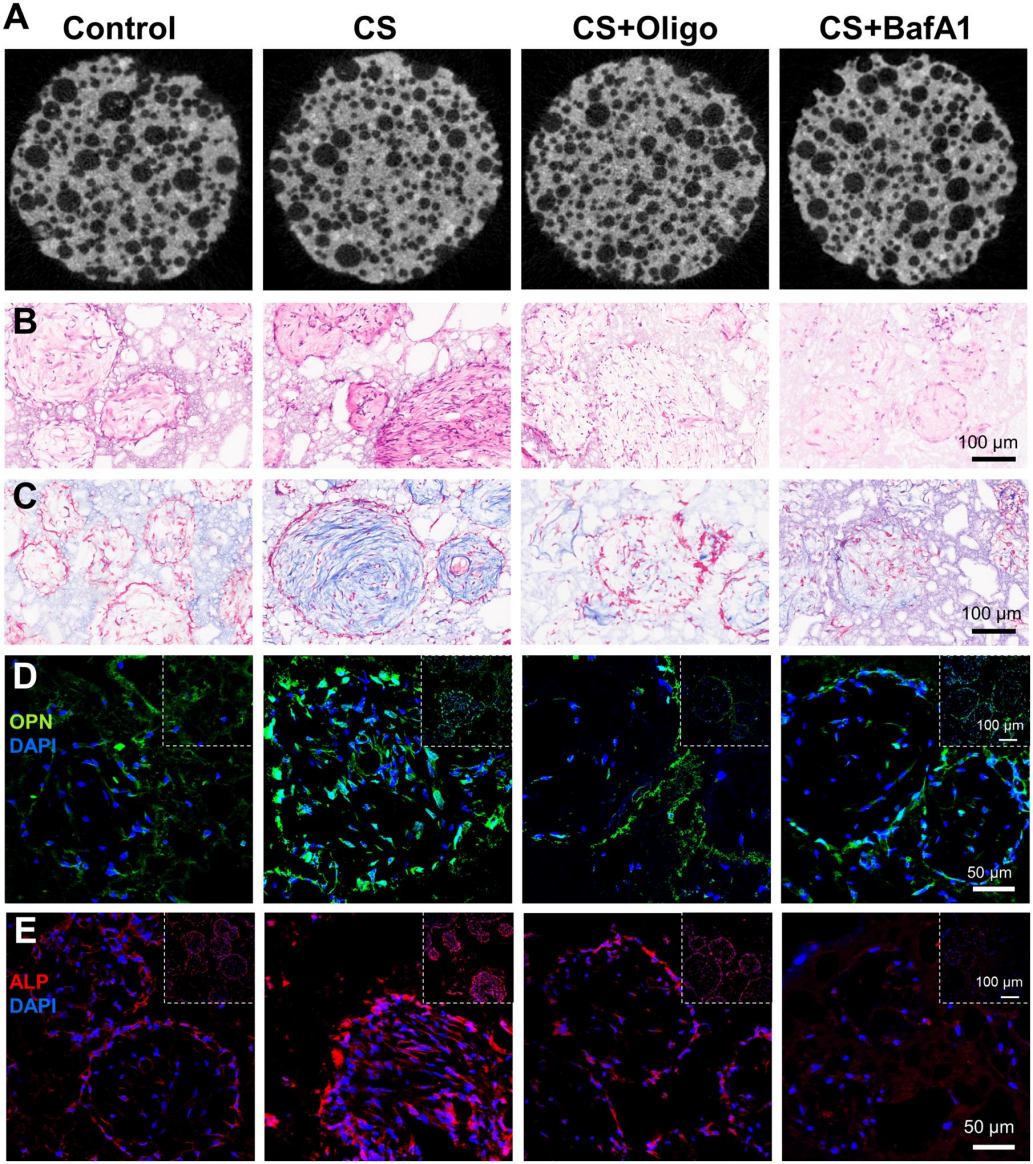
CS-induced OXPHOS enhances the osteogenesis of hBMSCs *in vivo*. (**A**) Micro-CT images of implanted HA/β-TCP scaffolds loaded with untreated hBMSCs (Control), CS-induced hBMSCs (CS), hBMSCs treated with CS and Oligo (CS+Oligo), and hBMSCs treated with CS and BafA1 (CS+BafA1). (**B**) HE staining in the different groups. (**C**) Masson’s trichrome staining. (**D, E**) Immunofluorescence staining of OPN and ALP in the different groups. The larger images are magnified views of the panels in the upper right.

We further confirmed whether CS-induced mitochondrial OXPHOS enhances the osteogenic differentiation of hBMSCs *in vivo*. hBMSCs, CS-treated hBMSCs or Oligo/BafA1+CS-treated hBMSCs were seeded onto HA/β-TCP scaffolds and then transplanted subcutaneously into nude mice. After 8 weeks, micro-CT scanning of the implanted scaffolds revealed more areas with a high density of dots in the scaffolds loaded with CS-induced hBMSCs than in the control scaffolds (Figure 6A), suggesting more mineralized tissue deposition. However, Oligo/BafA1+CS-treated hBMSCs failed to enhance mineralization deposition and displayed a density similar to that of control hBMSCs. In addition, more collagen fibers were found in the CS group (Figure 6B and C), as indicated by HE and Masson’s trichrome staining. Stronger immunofluorescence staining for the osteogenic markers ALP and OPN was also observed in the CS group compared with the control group (Figure 6D and E, and Supplementary Figure S5A). In contrast, collagen fiber formation was markedly inhibited in the group treated with CS+Oligo or BafA1 compared with the group treated with CS alone (Figure 6B and C). Similarly, immunofluorescence staining for OPN and ALP was impaired in the Oligo/BafA1+CS-treated hBMSC groups (Figure 6D and E, and Supplementary Figure S5A), demonstrating that CS significantly enhances the osteogenesis of hBMSCs *in vivo* and that the mitophagy-mediated metabolic transition toward OXPHOS plays a critical role in these processes.

**Figure 7.**
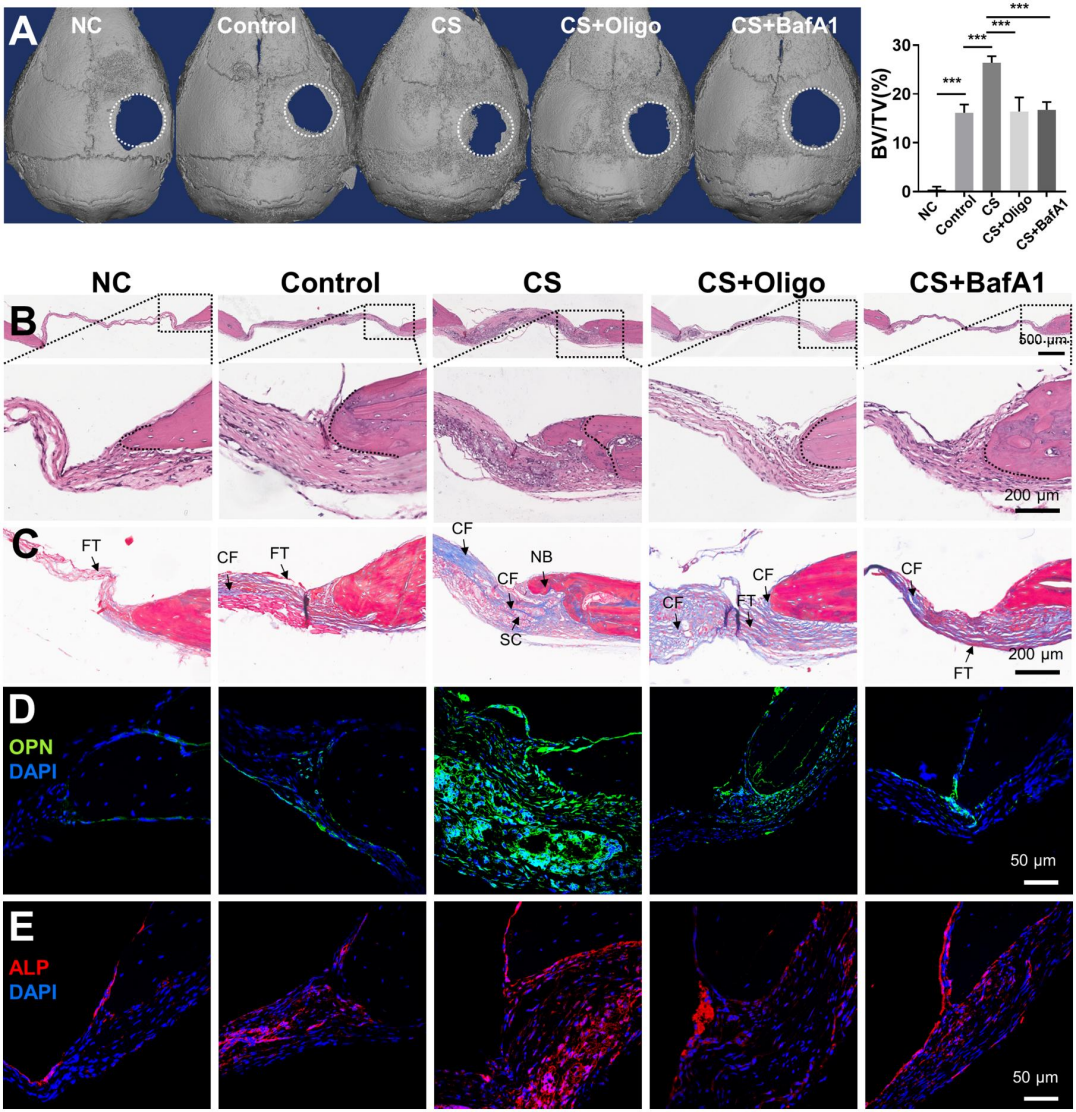
CS-induced OXPHOS improves the bone repair ability of hBMSCs *in vivo*. (**A**) 3D reconstructions of micro-CT images of mouse craniums and the BV/TV in each group (*n* = 5). Empty scaffolds (NC group), untreated hBMSCs (control group), CS-induced hBMSCs (CS group), hBMSCs treated with CS and BafA1 (CS+BafA1 group), or hBMSCs treated with CS and Oligo (CS+Oligo group) were implanted in the defects. (**B**) HE staining of defects in mice from the different groups. Higher magnification images (lower panels) of the regions outlined by the dotted lines in the lower magnification images (upper panels) are shown. (**C**) Masson’s trichrome staining in the multiple groups. (**D, E**) Immunofluorescence staining of OPN and ALP in mouse calvarial defect tissue. NB, new bone; SC, undegraded scaffold; CF, collagen fiber; FT, fibrous tissue.

A critical-sized calvarial defect mouse model was constructed to evaluate the bone repair potential of CS-treated hBMSCs. Micro-CT 3D reconstructions showed markedly enhanced bone regeneration in the CS-treated hBMSC group compared to the NC group (PLGA scaffold alone), with bone formation extending beyond the calvarial margin. In contrast, untreated hBMSCs induced only modest new bone formation. Corresponding BV/TV values displayed a significant elevation in the CS group compared to controls (26.14 ± 1.37% vs. 16.66 ± 1.83%, *P* < 0.001) (Figure 7A). New bone and collagen fiber generation in the bone tissue defect area were observed via HE and Masson’s trichrome staining. In the NC group, fibrous tissue made up most of the defect area, and few collagen fibers formed. Compared to the NC group, the scaffolds in the untreated BMSC group presented a small amount of collagen fibers among abundant fibrous tissues. Notably, scaffolds loaded with CS-induced hBMSCs exhibited extensive new bone and collagen fiber formation in the defect area and alongside the undegraded scaffolds (Figure 7B and C). We assessed the levels of the osteogenic hallmarks OPN and ALP via immunofluorescence staining. OPN-positive areas were barely observed in the NC group and untreated hBMSC group, whereas the CS-treated hBMSCs exhibited remarkable OPN and ALP expression, indicating active and robust osteogenesis. We also observed OPN- and ALP*-*positive cells, especially around the newly formed bone, indicating that osteoblasts lined the bone margins (Figure 7D and E, and Supplementary Figure S5B). These data confirm the enhanced osteogenic or bone repair capacity of CS-treated hBMSCs *in vivo.*

Next, we applied the OXPHOS inhibitor Oligo or mitophagy inhibitor BafA1 to CS-treated hBMSCs to further confirm that CS enhances the bone repair capacity of hBMSCs through mitophagy-mediated metabolic transition *in vivo.* New bone formation in calvarial defects was impaired in the groups implanted with hBMSCs exposed to CS and Oligo or BafA1 compared to the group implanted with hBMSCs treated with CS alone, as indicated by the micro-CT images in Figure 7A, together with the decreased BV/TV (26.14 ± 1.37% in the CS group vs. 16.83 ± 2.59% in the CS+Oligo group, *P *< 0.001; 26.14 ± 1.37% in the CS group vs. 17.02 ± 1.31% in the CS+BafA1 group, *P *< 0.001). Histological findings also revealed that the addition of Oligo/BafA1 largely diminished new bone and collagen fiber formation in CS-treated hBMSCs (Figure 7B and C) and led to weak OPN and ALP expression (Figure 7D and E, and Supplementary Figure S5B). These results further indicate that the enhanced osteogenic or bone repair capacity of CS-treated hBMSCs *in vivo* depends on the mitophagy-mediated metabolic transition toward OXPHOS.

## Discussion

CS-based biomaterials have been verified to exert osteoinductive effects on MSCs to accelerate the healing process in bone tissue engineering by modulating various signaling pathways (e.g. BMP, NF-kB, Wnt, Shh, and MAPK/ERK) in MSCs. Herein, we revealed that enhanced mitochondrial OXPHOS is critical for CS- or Si ion-induced osteogenesis in hBMSCs, providing novel insights into how CS-based bioactive materials promote the osteogenic differentiation of MSCs from a metabolic perspective. A recent study reported that supramolecular hydrogels with high network dynamics enhanced the osteogenic capacity of encapsulated MSCs by increasing tricarboxylic acid (TCA) cycle activity, OXPHOS, and ATP biosynthesis [5]. In another study, Mg^2+^ in methacrylated alginate microgels was shown to act as an “energy propellant”, significantly increasing the osteoinductivity of BMP-2 by activating glycolysis and OXPHOS to thereby meet the bioenergetic demand for osteogenesis [4]. A piezoelectric BaTiO_3_/Ti_6_Al_4_V (BT/Ti) scaffold enhanced OXPHOS and ATP synthesis in macrophages, modulating the immune microenvironment and triggering bone regeneration in a sheep cervical corpectomy model [30]. Likewise, MSCs encapsulated in a cell-adaptable hydrogel showed increased TCA cycle activity, OXPHOS, and ATP production via an E-cadherin- and AMP-activated protein kinase (AMPK)-dependent pathway, facilitating bone regeneration in a rat calvarial defect model [5]. These investigations indicate that metabolic regulation via biomaterials represents a promising osteogenic strategy for promoting bone regeneration, with metabolic reprogramming toward OXPHOS in MSCs emerging as a novel therapeutic approach to achieve superior clinical outcomes. The activation of mitochondrial OXPHOS supports the energy demands for the extensive biosynthesis of extracellular matrix proteins throughout the osteogenesis of BMSCs [20]. Metabolic intermediates of OXPHOS also directly influence MSC differentiation through epigenetic regulation [31]. Increased synthesis of acetyl donor acetyl coenzyme A (acetyl-CoA) from citrate during active OXPHOS enhances β-catenin acetylation and stabilization, leading to increased translocation to the nucleus. The nuclear translocation of β-catenin then increased the canonical Wnt target genes expression, including Runx2 and Osx, to induce osteogenic differentiation by binding with transcriptional coactivators [10, 32, 33]. Here, we found that CS enhanced the basal and maximal respiration capacity, and ATP generation, in hBMSCs. Furthermore, CS-treated hBMSCs presented an increase in citrate levels and a unique MSEA profile characterized by increased transfer of acetyl groups into mitochondria. The inhibition of OXPHOS by Oligo hindered CS-induced osteogenesis and bone regeneration. Thus, CS-induced OXPHOS may provide additional energy and certain metabolic intermediates (such as citrate) to enhance osteogenesis. The precise mechanisms need to be clarified by further research.

Mitochondrial dynamics, including coordinated cycles of fusion and fission, is a main contributor to the metabolic profile and MSC fate determination [34]. During osteogenic differentiation, MSCs exhibit increased mitochondrial fusion to form a network, which indicates mitochondrial activation and the formation of tightened cristae to maximize OXPHOS activity [8, 19, 22]. In this study, we demonstrated that CS-treated hBMSCs presented increased mitochondrial fusion, which may have helped promote OXPHOS activity to facilitate osteogenesis. However, earlier outcomes concerning the impacts of mitochondrial dynamics on the osteogenic differentiation of MSCs remain controversial. Mfn1 and Mfn2 expression, which mediate outer mitochondrial membrane fusion, was shown to increase throughout the early stages of osteogenic induction in hBMSCs and mouse skin MSCs (msMSCs), whereas the fission-related protein fission 1 (FIS1) expression remains unchanged [35, 36]. Knockdown of Mfn2 suppresses OXPHOS activity in msMSCs, thus leading to impaired osteogenesis [36]. Our results are consistent with these reports. In contrast, another study showed that inhibiting mitochondrial fission with Mdivi-1 results in decreased stemness and osteogenesis in rat BMSCs [37]. In addition, a recent study demonstrated that MFN2 suppression triggers the osteogenic differentiation of iPSC-MSC through Wnt/β-catenin pathway–mediated aerobic glycolysis, with no significant impact on OXPHOS [38]. These contrasting findings may be attributed to the severity of the stimuli or the degree of variation in mitochondrial dynamics.

A few studies have indicated that mitophagy drives metabolic reprogramming to glycolysis or OXPHOS based on the cell demand [29, 37, 39, 40]. Here, we found that CS induced mitophagy in hBMSCs by enhancing autophagic flux, facilitating the removal of dysfunctional mitochondria, promoting mitochondrial rejuvenation, and enhancing respiration to meet energy demands during bone regeneration. Autophagy, and potentially mitophagy, may recycle metabolites to power OXPHOS, as recent findings suggest that autophagy supports mitochondrial respiration by fueling one-carbon metabolism through serine availability [41]. However, prolonged mitophagy reduces mitochondrial mass or number, leading to a metabolic shift toward glycolysis [42, 43], suggesting that both the extent and duration of mitophagy influence metabolic transition. On the other hand, changes in metabolites of the TCA cycle subsequently affect mitophagy. An increase in citrate or acetyl groups caused by CS treatment might accelerate mitophagy through the dynamic acetylation of core autophagy proteins, thus resulting in positive feedback [44, 45]. Further research is needed to explore whether similar positive feedback exists. In addition, similar biomaterials can activate autophagy-related signaling pathways (e.g. mTOR and AMPK). Dicalcium silicate (C_2_S) nanoparticles can stimulate the mTOR/ULK1 pathway to trigger autophagy and then trigger the WNT/β-catenin pathway to increase the differentiation and biomineralization of osteoblasts [46]. C_2_S also regulates the expression of autophagy-associated proteins (PINK1, Parkin, Beclin1 and LC3) in macrophages [47]. Another study reported that a β-calcium silicate (β-CS)/PDLGA composite scaffold promoted BMSC osteogenesis via the stimulation of the AMPK/ERK1/2 and PI3K/Akt pathways [48]. These activated signals might result in enhanced mitophagy. Besides, in these previous studies, biomaterials were found to induce the LC3-II expression, illustrating elevated generation of autophagosomes and thus increased autophagy. However, in our study, CS treatment suppressed the expression of LC3-II and p62, and blocking autophagic flux via BafA1 resulted in increased and almost similar LC3-II and p62 expression in the control and CS-treated hBMSCs, suggesting that CS may accelerate lysosomal clearance but not the formation of autophagosomes. Consistently, TEM revealed a markedly increased number of autolysosomes in the CS-treated group, whereas few autophagosomes were present. Consequently, our investigation illustrated a distinct mechanism by which CS enhances mitophagy, possibly by accelerating the fusion of autophagosomes with lysosomes and lysosomal clearance. Nevertheless, additional investigation is required to examine the upstream molecular signals linking CS exposure to mitophagy.

In addition to the effects of mitophagy on metabolic shifts, mitophagy serves a pivotal function in modulating the mtROS levels, which are major sources of reactive oxygen species (ROS) [26]. Dysfunctional mitochondria usually exhibit Ca^2+^ overload, which increases ROS generation [49–51]. During the osteogenesis of MSCs and osteoblasts, ROS levels decrease [11,12]. Accumulated ROS impair the osteogenic differentiation of MSCs by inhibiting the Wnt/β-catenin and Hedgehog pathways, while activating the antioxidant defense system ameliorates the impairment of osteogenesis induced by oxidative stress [20, 52]. Mitophagy can limit the increase in ROS levels and protein oxidation resulting from a high-glucose environment to restore the osteoblastic MC3T3-E1 cell differentiation [53, 54]. In contrast, downregulation of the core regulator of mitophagy PINK1 can impair mitochondrial homeostasis and increase mitochondrial ROS production, thereby inhibiting osteoblast differentiation [55]. Recently, biomaterials that target mitochondria, such as Mn_3_O_4_@PDA@Pd-SS31 nanozymes and nanocatalytic metal–organic framework coatings, which directly activate mitophagy and inhibit ROS production, thus assisting in bone tissue regeneration, have been developed [56, 57]. In line with this, in our study, CS-induced mitophagy may have also inhibited mtROS generation to enhance the osteogenic and bone repair capacity of hBMSCs. In fact, mitophagy initiation can restore mitochondrial function and decrease ROS production in periodontal ligament stem cells (PDLSCs) in periodontitis, resulting in rescued osteogenic differentiation of PDLSCs and decreased periodontal bone loss [58]. Mitophagy also preserves the osteogenic differentiation of MSCs through other mechanisms, such as maintaining the stemness of MSCs [59].

## Conclusions

In summary, our results reveal that CS can induce OXPHOS to promote the osteogenic differentiation of hBMSCs, mainly through Si ion release. The induction of mitophagy by CS facilitates the maintenance of mitochondrial fitness to enhance OXPHOS. These findings offer valuable vision into the role of mitochondrial metabolism in promoting the osteogenesis of MSCs via the use of bioactive materials.

## Supplementary Material

rbaf101_Supplementary_Data
