# Supplementary material for: Calcium silicate induces mitophagy-mediated metabolic shifts toward oxidative phosphorylation in BMSCs to facilitate osteogenesis and bone regeneration
Source: Regen Biomater. 2025 Oct 1;12:rbaf101. doi: 10.1093/rb/rbaf101 (PMC12582392; doi:10.1093/rb/rbaf101)
Supplement: rbaf101_Supplementary_Data [file rbaf101_supplementary_data.zip › final-RB-2025-267.R2-Supplementary-data-修改.docx]

**Table S1. Primer sequence for RT-PCR**

| **Primers** | **Sequence 5’-3’** |
| --- | --- |
| ***Runx2*** | \| F: CCGCCTCAGTGATTTAGGGC \| \| --- \| \| R: GGGTCTGTAATCTGACTCTGTCC \| |
| ***Osx*** | \| F: CCGCCTCAGTGATTTAGGGC \| \| --- \| \| R: GGGTCTGTAATCTGACTCTGTCC \| |
| ***ALP*** | \| F: CGTCGATTGCATCTCTGGGCTCC \| \| --- \| \| R: TGGTCTCGCCAGTACTTGGGGT \| |
| ***Col-1*** | \| F: GAGGGCCAAGACGAAGACATC \| \| --- \| \| R: CAGATCACGTCATCGCACAAC \| |
| ***Fis1*** | \| F: AGCGGGATTACGTCTTCTACC \| \| --- \| \| R: CATGCCCACGAGTCCATCTTT \| |
| ***Drp1*** | \| F: CTGCCTCAAATCGTCGTAGTG \| \| --- \| \| R: GAGGTCTCCGGGTGACAATTC \| |
| ***Mtp18*** | \| F: GGTGGACACCTTTGTATGGCA \| \| --- \| \| R: GTCAATGGGGTGGATAATGATGG \| |
| ***Mfn1*** | \| F: TGGCTAAGAAGGCGATTACTGC \| \| --- \| \| R: TCTCCGAGATAGCACCTCACC \| |
| ***Mfn2*** | \| F: CTCTCGATGCAACTCTATCGTC \| \| --- \| \| R: TCCTGTACGTGTCTTCAAGGAA \| |
| ***β-actin*** | F: ATCAAGATCATTGCTCCTCCTG  R: GTCATACTCCTGCTTGCTGAT |

**
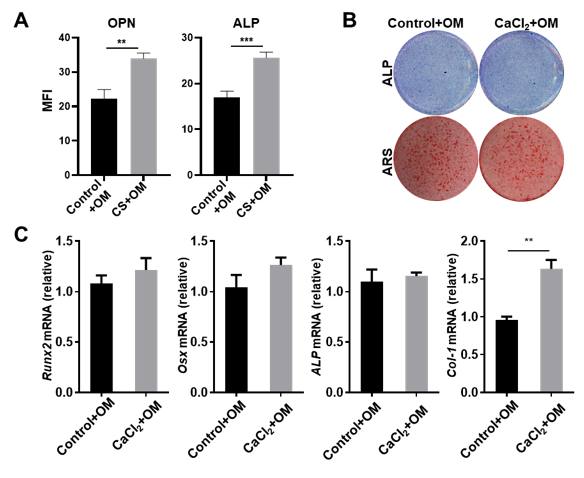
**

**Supplementary Figure S1.** CS-induced osteogenic differentiation maybe mainly *via* Si ion release instead of Ca ion. (A) Quantification of mean fluorescence intensity (MFI) of OPN and ALP in hBMSCs corresponding to Figure 1E. (B) ALP staining at 7 days after osteogenic induction and ARS staining at 14 days after osteogenic induction in hBMSCs treated with CaCl_2_ (69.4 mg/L). (C) qPCR analysis showed the relative mRNA levels of *Runx2*, *Osx*, *ALP* and *Col-1* in hBMSCs treated with CaCl_2_ at 7 days after osteogenic induction. Error bars represent the mean ± SD. ***P* < 0.01, ****P* < 0.001.

**
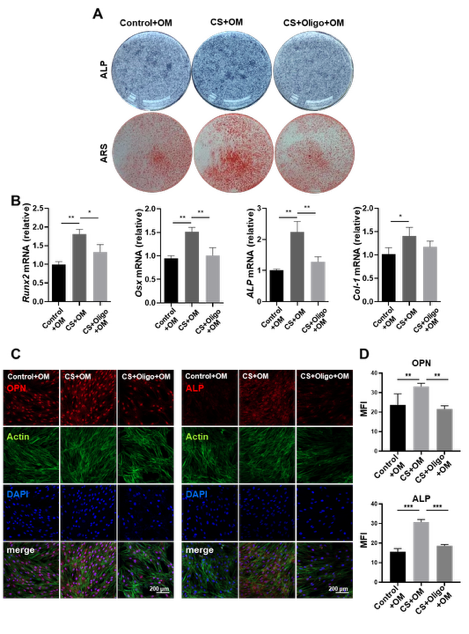
**

**Supplementary Figure S2.** CS-induced OXPHOS contributes to the osteogenic differentiation of hBMSCs. (A) ALP staining at 7 days after osteogenic induction and ARS staining at 14 days after osteogenic induction in hBMSCs treated with CS and oligomycin. (B) qPCR analysis showed the relative mRNA levels of *Runx2*, *Osx*, *ALP* and *Col-1* in hBMSCs treated with CS and oligomycin at 7 days after osteogenic induction. (C) Immunofluorescence staining of OPN and ALP in the hBMSCs treated with CS in the presence or absence of oligomycin at 4 days after osteogenic induction. (D) Quantification of MFI of OPN and ALP in hBMSCs. Error bars represent the mean ± SD. ****P* < 0.001; ***P* < 0.01; **P* < 0.05.


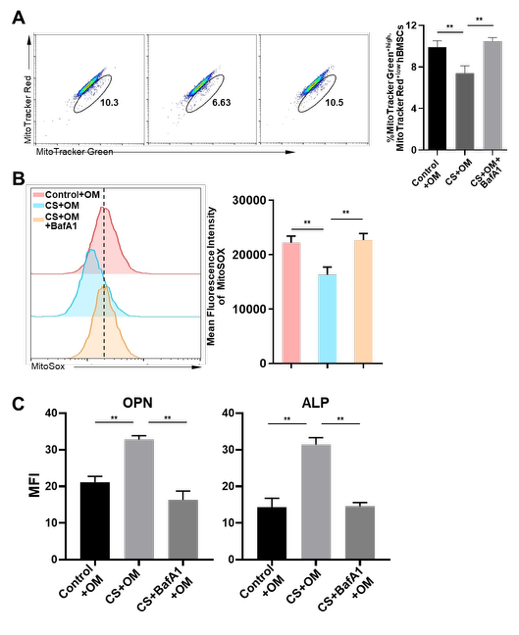


**Supplementary Figure S3.** CS eliminates dysfunctional mitochondria in hBMSCs in the presence of osteogenic medium (OM). (A) The hBMSCs stimulated with CS in the presence of BafA1 were labelled with MitoTracker Green and MitoTracker Red probes. The percentages of dysfunctional mitochondria were analysed. (B) The mtROS levels in hBMSCs stimulated with CS in the presence of BafA1 were assessed by MitoSOX fluorescence intensity. (C) Quantification of MFI of OPN and ALP in hBMSCs corresponding to Figure 5F. Error bars represent the mean ± SD. ***P* < 0.01.


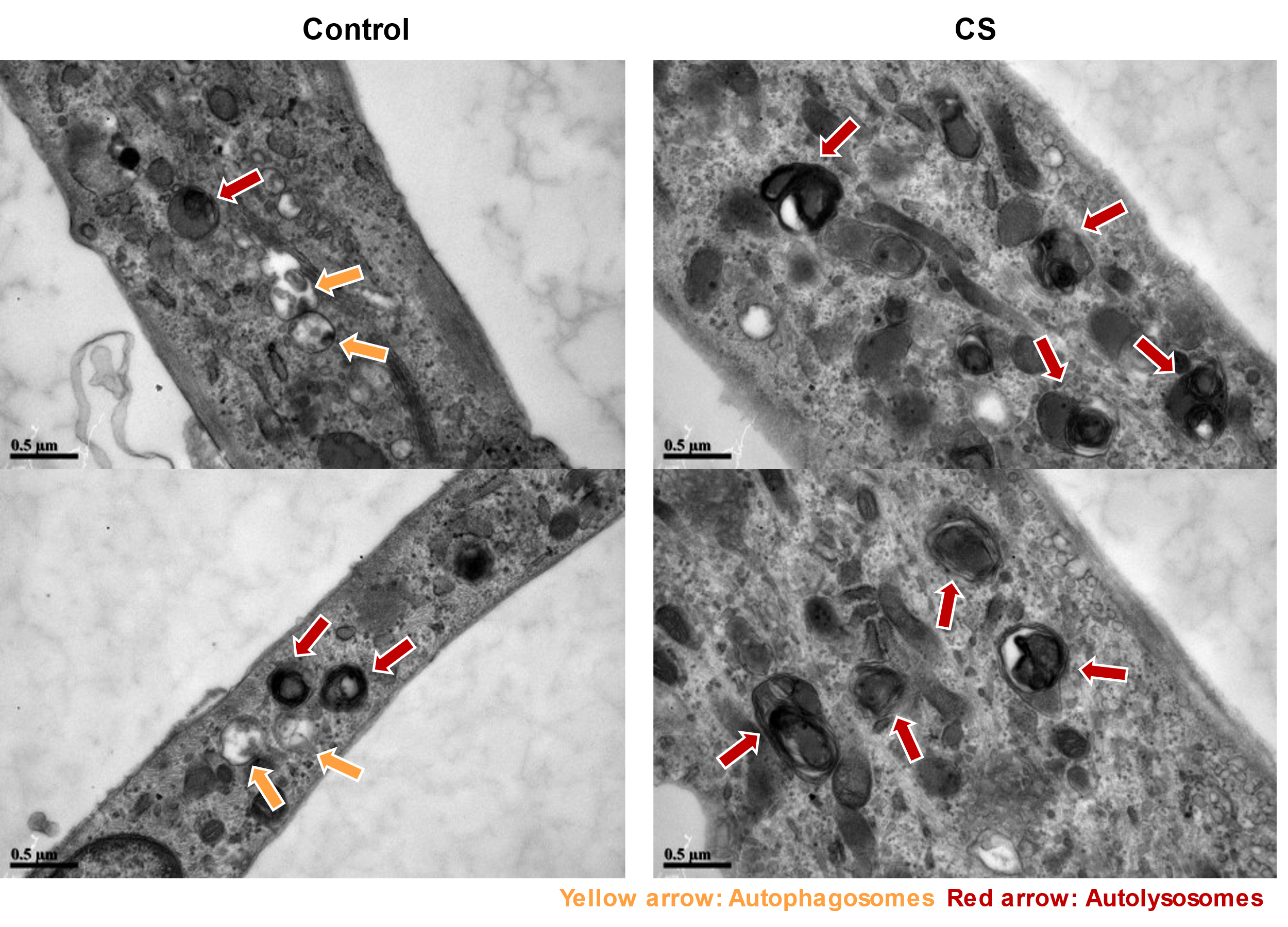


**Supplementary Figure S4.** Transmission electron microscope showed autophagosomes (orange arrows) and autolysosomes (red arrows) in control and CS-treated hBMSCs.

**
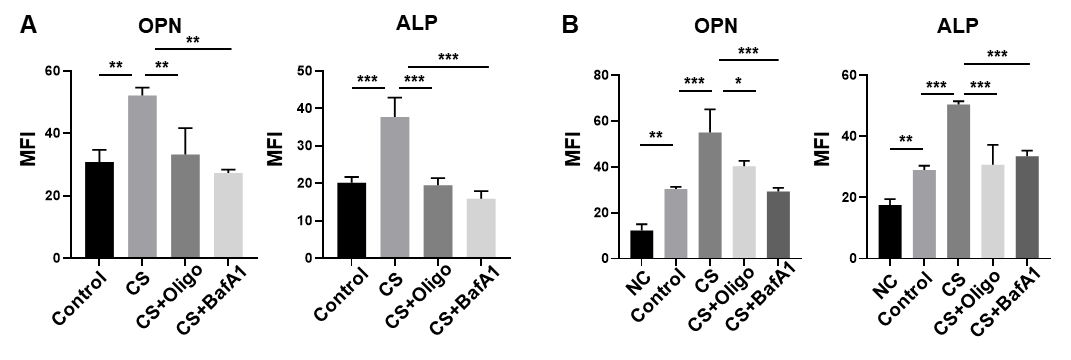
**

**Supplementary Figure S5.** Quantification of MFI of OPN and ALP in samples of (A) subcutaneous transplantation model and (B) critical-sized calvarial defect mouse model, corresponding to Figure 6D-E and 7D-E, respectively. Error bars represent the mean ± SD. ****P* < 0.001; ***P* < 0.01; **P* < 0.05.


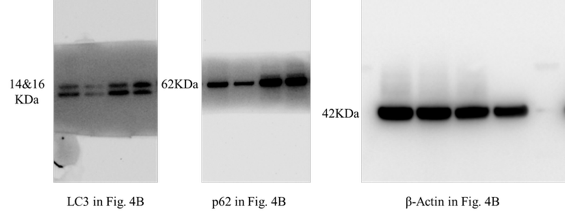


**Supplementary Figure S6.** The uncropped full-length gels and blots in Fig. 4B.
